# Medically Refractory Multiple Sclerosis Is Successfully Treated with Plasmapheresis in a Super Morbidly Obese Pregnant Patient

**DOI:** 10.1155/2020/4536145

**Published:** 2020-04-04

**Authors:** Lindsey Dalka, Antoine Harb, Kael Mikesell, Gillian Gordon Perue

**Affiliations:** ^1^Eastern Maine Medical Center, Department of Family Medicine, Bangor, USA; ^2^Eastern Maine Medical Center, Department of Hematology and Oncology, Bangor, USA; ^3^Eastern Maine Medical Center, Department of Patient Blood Management, Bangor, USA; ^4^Eastern Maine Medical Center, Department of Neurology, Bangor, USA

## Abstract

Multiple sclerosis (MS) is a relapse remitting immune-mediated demyelinating neurological disorder that primarily affects women of childbearing age. In most patients, the hormonal changes during pregnancy are protective against MS relapses. When relapses do occur, treatment options are limited to use of intravenous steroids and plasmapheresis rescue therapy. We present a case of steroid refractory MS-transverse myelitis with quadriplegia in a 25-year-old pregnant super morbidly obese woman. Our clinical case is unique because the severity of her relapse early in pregnancy, which was intractable and resistant to steroids. This may have been a rebound demyelination due to the discontinuation of fingolimod; a newly recognized entity by the FDA. Our case report therefore seeks to raise awareness about a potential complication of discontinuing MS disease modifying therapies, highlighting that these rebound relapses can be steroid resistant and occur despite the usual protective hormonal influence of early pregnancy and that plasma exchange is a valid treatment option. Finally, we discuss the challenges of determining exchange volumes for plasmapheresis in the super morbid obese population to secure good maternal and fetal outcomes.

## 1. Introduction

Multiple sclerosis (MS) is an immune-mediated demyelinating neurological disorder that primarily affects women of childbearing age. Prior to the 1998 PRIMS study [1, 2], pregnancy in patients with MS was discouraged, fearing acute exacerbations, and poor maternal/fetal outcomes. With disease modifying treatments available and reassuring registry data, pregnancy in patients with MS is now common.

The hormonal changes of pregnancy are usually protective against exacerbations in MS. When exacerbations occur, they tend to be infrequent, mild, short lived, and responsive to intravenous methylprednisolone. Plasmapheresis is reserved for rescue therapy [3, 4]. However, rescue plasmapheresis is not well studied in pregnancy, and there are no studies on plasmapheresis in pregnancy with super morbid obesity as a comorbidity. We therefore present a case of a 25-year-old with severe disabling MS exacerbation, super morbid obesity in pregnancy successfully treated with plasma exchange.

## 2. Case Description

A 25-year-old G2 P0010 Caucasian female with past medical history of super morbid obesity (BMI 55.2), depression, and multiple sclerosis presented to us with bilateral lower extremity weakness and right-sided flank pain. At the time of presentation, she was at 18 weeks' gestation.

She was diagnosed with multiple sclerosis at age 17 after she developed right optic neuritis. Over a 3-year period, she tried multiple modifying agents including interferon beta 1-b which was associated with ovarian cyst formation requiring surgery and interferon beta 1-a, which had intolerable side effects and glatimer acetate. While on glatimer acetate, she had 2 relapses in 6 months. She was eventually transitioned to fingolimod with a single relapse shortly after initiation of therapy and treated with a 5-day course of solumedrol. Fingolimod was used successfully for 5 years and was discontinued 4 weeks prior to conception. Her pregnancy was complicated by a threatened abortion and persistent vaginal bleeding. She was placed on bed rest. During bed rest, she developed fever, flank pain, and severe paraparesis requiring hospitalization. She was diagnosed with an acute MS flare in the setting of pyelonephritis.

Antibiotics and intravenous methylprednisolone 1,000 mg daily were initiated. A noncontrast MRI of the brain and cervical spine revealed a new extensive demyelinating lesion from C1 to C4. After the completion of 6 days of intravenous (IV) methylprednisolone, her lower extremity function had moderately improved, and she was able to ambulate with a walker and was discharged to an acute inpatient rehabilitation center.

Three weeks later, she re-presented to the hospital with right-sided flank pain, generalized weakness resulting in a fall, and a tingling sensation in her extremities. Given the early recurrence, neurology was consulted. In the neurological exam, mental state was normal, pupils were normal symmetrical, and reactive to light. There was no facial asymmetry and the other cranial nerves were normal. Muscle tone was increased in her left upper extremity and bilateral lower extremities. In the motor exam, there was an asymmetrical moderate/severe tetraparesis mainly in the lower limbs and more pronounced distally. There was generalized hyperreflexia with bilateral clonus and bilateral Babinski. Sensation was impaired with decreased sensation to light touch, pinprick, and vibration without a clear sensory level.

**Figure 1:**
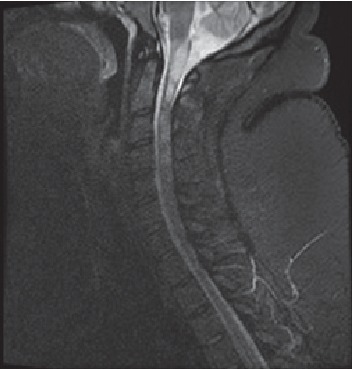
T2 sagittal flair image demonstrating an acute multiple sclerosis flare with longitudinally extensive transverse myelitis extending from the medulla to the upper cervical cord C3.

Urinalysis was consistent with a urinary tract infection and a urine culture grew out pan-sensitive *Escherichia coli*. A new MRI of the head (Figure 1) showed progression T2 hyperintense signal abnormality in the craniocervical cord. To exclude other differentials, her vitamin B12 and ACE levels were checked and were within normal limits. To rule out a possible diagnosis of neuromyelitis optica, an aquaporin 4 AQR4 antibody was tested and was negative.

The patient was started on antibiotics for suspected pyelonephritis, which was eventually changed to oral amoxicillin after urine culture sensitivities resulted.

The patient was started on IV methylprednisolone 250 mg every 6 hours. After five days of steroid treatment, she showed minimal motor improvement. At this time, rescue plasmapheresis (TPE) was initiated. Five treatments of TPE were performed every other day, alternating treatment days with IV methylprednisolone. The patient tolerated her therapy well and had progressive improvement in her neurologic symptoms with near complete resolution of her tetraparesis and mild residual sensory loss in her left arm. She was discharged to the acute rehabilitation service.

For the reminder of her pregnancy, she was treated with methylprednisolone 1000 mg as a single infusion every 3 weeks. She presented to the hospital at 36 weeks' gestation with contractions, vaginal spotting, hypertension, headache, and lower extremity edema. She was taken to the operating room for a cesarean section due to concerns of severe pre-eclampsia and preterm labor. She delivered a 2495 g male with APGARs of 8 and 9. After delivery, fingolimod was reinitiated in the outpatient setting.

## 3. Discussion

Our case report therefore seeks to raise awareness about the potential complication of rebound demyelination due to the discontinuation of fingolimod, a newly recognized entity by the FDA [5], highlighting that these rebound relapses can be steroid resistant and occur despite the usual protective hormonal influence of early pregnancy and that plasma exchange is a valid treatment option. In the case of fingolimod cessation for pregnancy, scheduled IV methylprednisolone for maintenance may be considered.

Treatment with TPE rescue provided unique challenges. While TPE has been used to successfully treat pregnant patients with other diseases [6–8], to the best of our knowledge, our case is only the third report of TPE for acute MS exacerbation in pregnancy [9, 10]. In the previous two case reports, the Nadler formula was used to determine the patient's blood volume (BV) and plasma volume (PV). Once the PV was determined, a plasma exchange of 1.5 PV was used to account for the 45–55% increase in PV that occurs starting in the second trimester of pregnancy [11, 12].

A unique feature of our case report is the size of our patient, as she weighed 152.2 kg when plasmapheresis was initiated. Using the Nadler formula and her hematocrit of 29.8, her PV calculated to 4841 mL. If a 1.5 PV exchange was performed (accounting for pregnancy), this would have resulted in a 7262 mL exchange. An exchange of this volume would have put the patient and fetus at increased risk for complications and may be unnecessary. While PV increases with weight, it does not do so in a linear fashion [13]. A morbidly obese patient has more adipose tissue which requires less blood supply. As a result, the Nadler formula may give an erroneously elevated BV in the morbidly obesity. One solution to overcome this miscalculation would be to use an adjusted body weight with the Nadler formula and then perform a 1.5 PV exchange based on the result. Alternatively, one could use a 1 PV exchange using the patient's actual body weight for the Nadler formula. Using the latter for our case led to 5000 mL exchange. TPE was successfully completed and improved the patient's symptoms appropriately. During TPE, the patient was positioned on the left lateral decubitus position to prevent inferior vena cava pressure and maximize venous return [9]. In addition, CMP and fibrinogen were monitored prior to each treatment to ensure that proper coagulation and calcium level was achieved. This method is safe and effective with good outcomes for both mother and child.

This case demonstrates that in patients with treatment refractory multiple sclerosis in pregnancy or fingolimod rebound demyelination therapeutic plasma exchange is safe and successful and that super morbid obesity should not be a contraindication. We hope that this will encourage future practitioners who face this dilemma to consider plasma exchange.
